# Increasing Blood Glucose Variability Is a Precursor of Sepsis and Mortality in Burned Patients

**DOI:** 10.1371/journal.pone.0046582

**Published:** 2012-10-09

**Authors:** Alexander N. Pisarchik, Olga N. Pochepen, Liudmila A. Pisarchyk

**Affiliations:** 1 Centro de Investigaciones en Optica, Leon, Guanajuato, Mexico; 2 Belarus Medical Academy of Postgraduate Education, Minsk, Belarus; 3 Department of Medical Sciences, University of Guanajuato, Leon, Mexico; Technical University of Madrid, Italy

## Abstract

High glycemic variability, rather than a mean glucose level, is an important factor associated with sepsis and hospital mortality in critically ill patients. In this retrospective study we analyze the blood glucose data of 172 nondiabetic patients 18–60 yrs old with second and third-degree burns of total body surface area greater than 30% and 5%, respectively, admitted to ICU in 2004–2008. The analysis identified significant association of increasing daily glucose excursion (DELTA) accompanied by evident episodes of hyperglycemia (>11 mmol/l) and hypoglycemia (<2.8 mmol/l), with sepsis and forthcoming death, even when the mean daily glucose was within a range of acceptable glycemia. No association was found in sepsis complication and hospital mortality with doses of intravenous insulin and glucose infusion. A strong increase in DELTA before sepsis and death is treated as fluctuation amplification near the onset of dynamical instability.

## Introduction

A human organism, as a very complex dynamical system, is characterized by various variables and parameters, and as in any real system, a certain level of noise always presents. It is known that all physiological processes, including glucose metabolism, are accompanied by random fluctuations [1]–[3], which are thought to arise from the action of a large number of variables. Actually, the interaction between stochasticity and nonlinearity is one of the main issues in the study of dynamical systems, let it be, optical [4], climatic [5], populational [6], geophysical [7], or epidemical [8]. In physiology, an important source of noise was found in conductance fluctuations of ionic channels due to random times at which they open and close, although there are many other sources associated with channels [9].

During its life, any organism passes through at least two critical points – birth and death, the points where the system changes its stability. Yet in 1985 Wiesenfeld and McNamara [10], [11] hit upon an idea that a dynamical system near the onset of a dynamical instability (critical point) might be very sensitive to coherent or random perturbations. They showed that both periodic and random fluctuations are greatly amplified in the vicinity of different bifurcations, such as saddle-node, transcritical, pitchfork, period doubling, and Hopf. In the case of random fluctuations this phenomenon is referred to as *prebifurcation noise amplification*. According to the Wiesenfeld€s theory [10], prebifurcation noise amplification can serve as a precursor of a bifurcation in a nonlinear dynamical system; the closer to the critical point the system gets, the stronger noise amplification.

Many theoretical works have predicted generality of prebifurcation fluctuation amplification. For example, small-signal amplification was found near period-doubling bifurcations in smooth iterated maps [12], noise amplification close to a bifurcation point was identified as the physical mechanism behind dynamical instabilities in minority games with discounting [13]. It is indeed of great practical importance to know how far a system is from a critical point, knowing that strong changes in noise often anticipate many oncoming natural and biological catastrophes, like earthquakes, epidemics, population extinction, myocardial infarction, etc., and thus can serve as a precursor of the adverse natural phenomena or diseases.

Experimental evidence of the precursors of dynamical instabilities was reported in different areas. In medicine, the analysis of electroencephaloglams was performed to predict epileptic seizures [14]. In optics, both small-signal and noise amplifications were observed in lasers close to critical points [4], [15]. In hydrodynamics, precursors excited by deterministic perturbations were also found [16]. In geophysics, the analysis of direct data of seismic activity and electromagnetic anomalies were used to predict earthquakes [17].

In this work, we study blood glucose (BG) dynamics in heavily burned patients. In most previous studies, the typical BG measurements were glucose at admission, maximum glucose, and mean glucose levels. However, if an arithmetic mean is exploited for all measurements, it may falsely show normoglycemia, while actually hyperglycemic and hypoglycemic episodes are apparent [18]–[20]. In contrast to the widely accepted opinion that increased mortality in severely burned patients is associated only with hyperglycemia, hypoglycemia is also evident in patients with sepsis and at death risk [21], [22]. Only recently the importance of glucose variability was recognized as an independent factor associated with increasing mortality in diabetic [23], [24] and critically ill patients [25]. Latest studies provided the evidence that BG fluctuations are important and should be taken into account. High glucose excursion (>50% of values outside 4–6 mmol/l) was associated with increased mortality in diabetic [26]–[29], severely burned [30], and other critically ill patients [32]–[38].

The glucose variability includes periodic and random components [39], which is hardly possible to separate. The periodicity is associated with daily biorhythms and alimentation, while the random component or physiological noise is mainly determined by a current organism state. Among many measures of glucose variability [39], [40], the commonly used ones are the standard deviation (SD) from the mean of glucose measurements and the coefficient of variation (SD normalized to the mean) [41]. In this work, we examine the daily glucose excursion (DELTA), i.e., the difference between maximum and minimum glucose levels recorded during a day [42], [43]. Being similar to daily SD, DELTA is more sensitive variable, since this is the maximum amplitude of daily glucose fluctuations including periodic and random components. Through the analysis of clinical data we will show here that DELTA is a good precursor of the illness complication; its amplification indicates that the organism, as a complex dynamical system, approaches a critical point.

## Materials and Methods

### Setting and patients

In this analysis we included the data of 172 patients of 18–60 yrs with greater than 30% of total body surface area (TBSA) second-degree and greater than 5% third-degree burns without pre-burns diabetes mellitus history, admitted to the ICU of the Belarus Republic Burn Centre in 2004–2008. The clinical characteristics of the patients enrolled in this study are included in Table 1. The mean age was 38.3 (18.1–57.2) and 78% of the patients were male. The patients who died during a burn shock (first 1–2 days) have been excluded from this research because of a few data points to retrieve glucose dynamics. All patients were resuscitated according to the Parkland formula using the Ringer-Lactat solution. Within the first 48 hrs after admission all patients underwent wound excision (10–20% of TBSA, or when possible, total wound excision). The immediate wound coverage used autografts or frozen cadaver skin. When donor sites healed, the patients were taken back to the operating room every 5–10 days for replacement of cadaver skin to autografts.

**Table 1:** Comparison of clinical characteristics and glucose, cortisol, and insulin indices of three ICU groups: survivors without sepsis (Group 1), survivors with sepsis (Group 2) and non-survivors (Group 3).

Characteristic	Group 1	Group 2	Group 3	P_12_ 1	P_23_ 2
Number of patients	45	77	50		
Age, yr	33.7 [18.1–45.3]	38.8 [19.5–52.2]	42.3 [32.3–57.2]	<0.05	<0.05
ICU stay (days)	8.5 [5–14]	22.8 [21–31]	14.5 [12–27]	<0.05	<0.05
TBSA burn (%)	33 [23–56]	43.5 [35–48]	51.2 [45–58]	>0.05	<0.05
3rd degree burn (%)	8 [5–13]	21.4 [12–42]	38.3 [25–44]	<0.05	<0.05
APACHE II score (first 24 hrs)	13 [12–15]	17 [14–19]	18 [15–19]	<0.05	>0.05
Inhalation trauma patients (%)	15 (33.3%)	45 (58%)	31 (62%)	<0.05	>0.05
Artificial ventilation patients (%)	15 (33.3%)	58 (75.1%)	50 (100%)	<0.05	<0.05
Admission glucose (mmol/l)^3^	8.3 [7.8–9.2]	9.1 [7.1–10.5]	9.4 [6.8–12.1]	>0.05	>0.05
Admission cortisol (nmol/l)	677 [640–820]	780 [650–950]	740 [720–930]	<0.05	>0.05
Admission insulin (pmol/l)	444 [260–760]	240 [170–279]	156 [110–178]	<0.05	<0.05
Admission ScvO_2_ (%)	44.5 [42–48]	40.8 [35–42]	41.6 [36–43]	>0.05	>0.05
Glucose intake (g/24 hrs)	280 [180–325]	320 [240–400]	320 [280–380]	<0.05	<0.05
Insulin intake (IU/24 hrs)	64 [60–72]	100 [85–200]	114 [80–220]	<0.05	>0.05
Mean daily glucose^4^ (mmol/l)	5.6 [5.3–5.7]	6.6 [6.2–7.1]	8.1 [7.2–8.8]	<0.05	>0.05
Minimum daily glucose^4^ (mmol/l)	3.5 [3.1–3.8]	3.1 [2.3–3.3]	2.7 [2.1–3.1]	<0.05	<0.05
Maximum daily glucose^4^ (mmol/l)	7.7 [6.2–7.7]	14.7 [11.7–17.0]	15.2 [11.1–18.0]	<0.05	>0.05
**DELTA** 4 **(mmol/l)**	**2.1 [1.3–2.6]**	**4.3 [2.2–5.8]**	**7.2 [4.0–8.1]**	**<0.05**	**<0.001**
Minimum daily glucose (mmol/l)		3.2 [2.2–4.3]^5^	3.5 [1.3–5.2]^6^		<0.05
Maximum daily glucose (mmol/l)		11.6 [8.2–18.2]^5^	15.9 [10.2–25.0]^6^		>0.05
**DELTA (mmol/l)**		**8.1 [6.6–8.6]** 5	**11.2 [9.0–14.1]** 6		**<0.001**

Data are numbers (%) or medians [interquartile range]. APACHE = Acute Physiology and Chronic Health Evaluation; ICU = Intensive Care Unit, IU = International Units.

1 P_12_ value was determined with the use of Mann-Whitney U-test between survivors without sepsis and survivors with sepsis.

2 P_23_ value was determined with the use of Mann-Whitney U-test between survivors with sepsis and non-survivors.

3 To convert the values for glucose from mmol/l to mg/dl, multiply by 18.018.

4 During the whole ICU stay.

5 One day before a date when sepsis was identified.

6 One day before death.

### Blood glucose measurements

Whole blood samples were taken from central lines four times per day (at 6, 12, 18, and 22 hrs) and analyzed with the bloodgas/blood glucose analyzer StatProfile CCX NovaBiomedical (USA). Serum cortisol and insulin levels were measured by radioimmunoassay (Multi Kristall Gama Counter LB2111, Berthold Texnologies GmbH&Co). Sepsis was defined as a blood culture identifying the pathogen during hospitalization or at autopsy, in combination with leucocytosis or leucopenia, hyperthermia or hypothermia, and tachycardia. The patients were fed enterally as soon as possible. The data was connected with TBSA, third-degree burns, APACHE€II score, the length of ICU stay, cortisol and insulin levels, doses of intravenous glucose and insulin (per day) intake, the central venous oxygen saturation (ScvO_2_)*_i_* (from superior vena cava). For estimation and illustration of individual glucose fluctuations, all registered absolute glucose values of all patients were plotted versus time (hrs after the burn trauma) during all ICU period.

### Glucose-insulin-potassium solution (GIPS) protocol

On the second day of admission the following continuous GIPS was used: glucose 0.1 g/kg/h+insulin 0.025 IU/kg/h and 7.5% potassium chloride (2 mmol/kg/day), on the third day basically GIPS included glucose 0.15–0.20 g/kg/h+insulin 0.02–0.04 IU/kg/h and 7.5% potassium chloride (2 mmol/kg/day). The study in Leuven (Belgium) targeted a normal BG level to be 4.4–6.1 mmol/l [44], however, some other studies have hypothesized that 6.1 mmol/l is not the best target [45]. To achieve the BG target level of 3.5–8.0 mmol/l, GIPS was titrated intravenously with a perfusor according to the BG level. If the glucose level was lower than 3.1 mmol/l, glucose with potassium without insulin was titrated. When BG was between 8 and 11 mmol/l, the insulin infusion rate was 0.08 IU/kg/h (about 7–8 IU/h for person). When BG felt down below 8 mmol/l, the insulin infusion rate decreased on 2 IU/h every hour up to 2 IU/h. When BG grew up above 11 mmol/l, the insulin infusion rate increased up to 10 IU/h, however the glucose infusion rate was never below 0.1 g/kg/h.

### Statistical analysis

In our analysis, we separate the patients into three groups depending on the illness outcome: survivors without sepsis (Group 1), survivors with sepsis (Group 2), and non-survivors (Group 3) (died with sepsis and severity of single and multiple organ dysfunction). Variables within each group were summarized either as numbers (%) or as medians [25th–75th percentile] as appropriate. Multiple regression analysis, Bravais-Pearson correlation coefficient (r), sensitivity and specificity were used as statistical methods. Statistical comparisons between groups were made using the Kruskal-Wallis ANOVA and Median test; P <0.05 was considered statistically significant (STATISTICA 6 and ORIGIN 7).

### Ethics statement

This study was approved by the Research Ethics Committee of the Belarus Medical Academy of Postgraduate Education. The medical data was taken from the Burn Center of the Republic of Belarus, where one of the authors, O. N. Pochepen, acting as the Head of the Burn Center of the Emergency Hospital in Minsk, had the authority to provide us with the data. All surviving patients, before leaving the hospital, signed an agreement for their medical data, including the data of their blood analyses, to be used anonymously in future research. The Research Ethics Committee gave its approval to use all data, including that of non-surviving patients, for this specific research. All data were analyzed anonymously.

## Results

**Figure 1:**
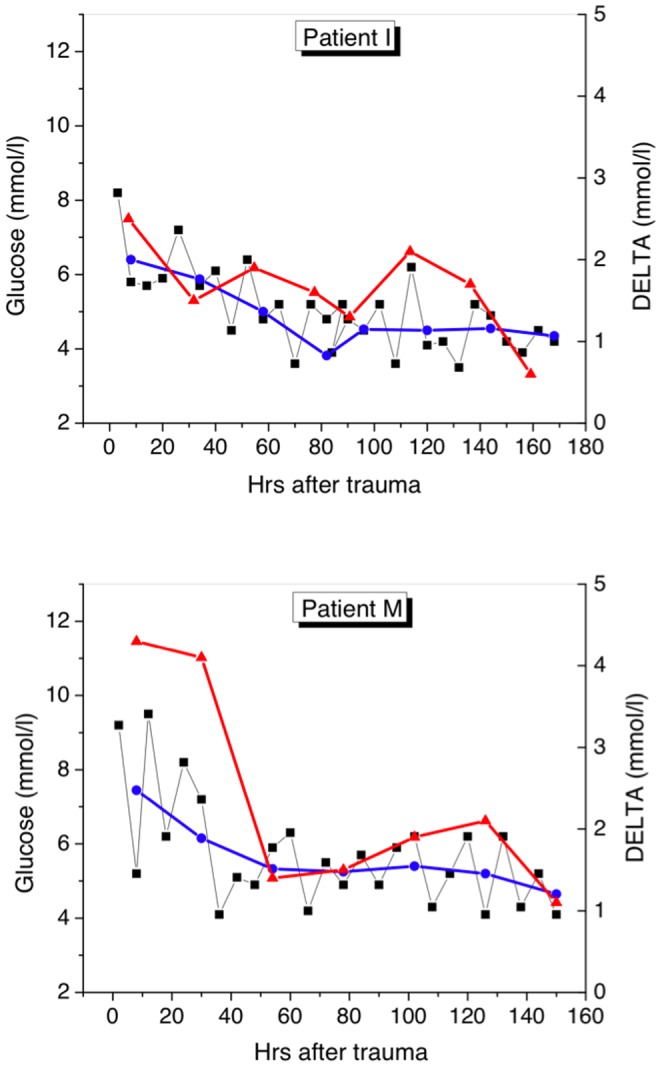
Time series of glucose measurements for patients from Group 1 (survivors without sepsis). Instant BG concentration (left axis), mean daily glucose (left axis), and daily glucose variation DELTA (right axis) are shown, respectively, by black squares, blue dots, and red triangles.

**Figure 2:**
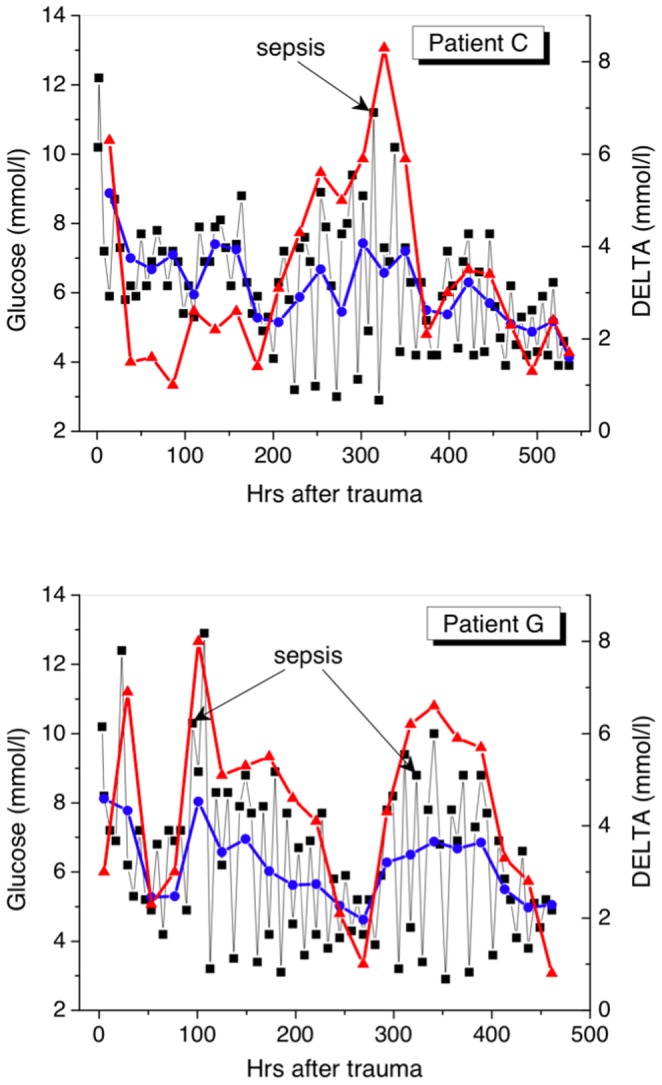
Time series of glucose measurements for patients from Group 2 (survivors with sepsis). The arrows indicate the measurements in which the blood culture was identified. DELTA reaches a maximum value in a sepsis state. Patient G had two sepsis episodes during his ICU stay.

**Figure 3:**
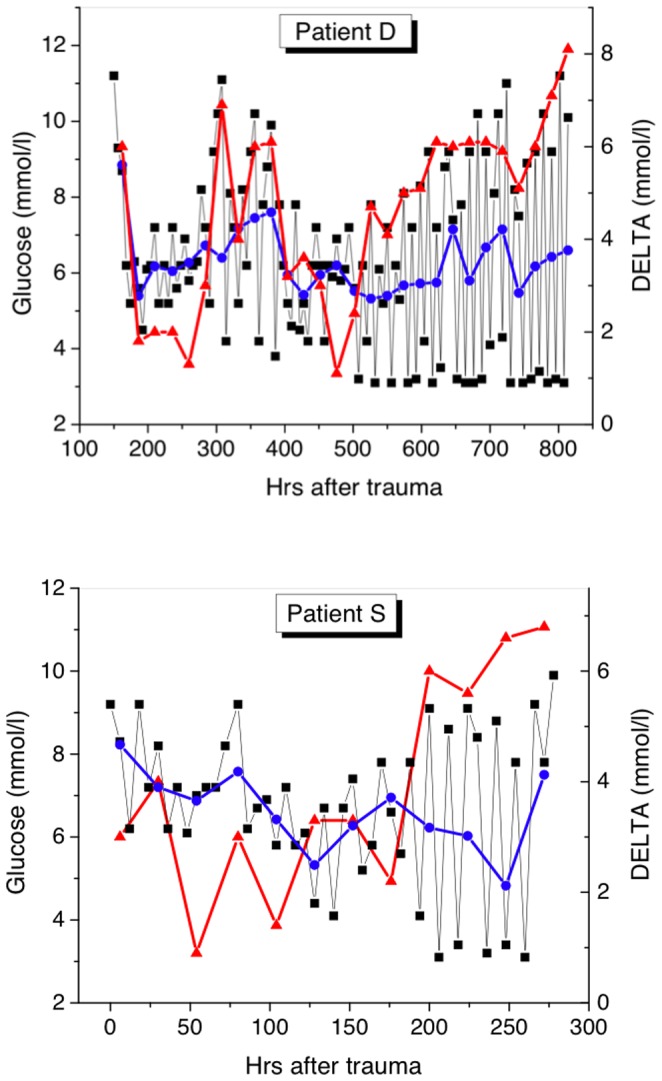
Time series of glucose measurements for patients from Group 3 (non-survivors). DELTA increases drastically before death, while the mean daily glucose remains within an acceptable range.

Table 1 lists clinical characteristics and glucose-related measures for patients belonging to every group during their ICU stays. The representative time series of the absolute glucose concentration (squares) and DELTA (red triangles) for two patients from each group are shown in Figs. 1, 2, 3. The daily mean glucose is plotted in the same figures with blue dots. Let us consider common and particular features inherent to each group.

### Group 1 (survivors without sepsis)

In this group of survivors without sepsis, the blood culture was negative, while hyperglycemia was present at admission and lasted only one day, that is associated with high cortisol (677 (640–820) nmol/l) (r = +0.35) and insulin (444 (260–760) pmol/l) (r = +0.38) levels. In the first 6–8 hrs after admission all patients had hypovolemia (CVP = 1–2 cm H_2_O) compensated by a high peripheral pressure with a high mean arterial pressure (111.8 (110–115) mm Hg). Acute stress hyperglycemia [46], [47], the typical reaction to stress, took place in the acute period of the burn injury (first one-three days, so-called “ebb phase”) and during sepsis as well when endocrine changes characterized by an unbalanced shift between insulin and counter-regulatory hormones occurred [48]. We suppose that hyperglycemia could be a consequence of depressed tissue oxygenation, decreased regional blood flow (ScvO_2_ = 44.5 (42–48)%), and reduced glucose utilization. During that period, both hypothermia and oliguria were clinically detected. On the second day, after resuscitation of the hemodynamic failure, mean glycemia was 5.6 (4.2–7.3) mmol/l. In the “ebb phase” the maximum glucose level reached up to 9.7 (8.2–10.2) mmol/l, and DELTA was 3.8 (2.2–4.0) mmol/l, whereas in the consecutive “flow phase” both the mean glucose and DELTA stabilized (Fig. 1).

### Group 2 (survivors with sepsis)

The survival with sepsis patients had higher TBSA and higher APACHE II in first 24 hrs than the patients from the first group. 45 (58%) patients had inhalation injury and were intubated in the first day; 13 patients were intubated when severe sepsis was identified. For this group, glucose at admission and ScvO_2_ were very close to those for Group 1, while the cortisol level was a little higher and the insulin level was lower. In the “flow phase”, three days after trauma, mean, maximum, and DELTA were higher than for Group 1, while the minimum level was lower. As a rule, a positive blood culture was detected two-three days after DELTA began to grow up (Fig. 2). In all patients, in the day when sepsis was identified DELTA exceeded 6 mmol/l [Fig. 2]. Some severely burned patients suffered from sepsis more than once during the ICU stay, for example, the patient G [see Fig. 2(b)]. In sepsis, hyperglycemia was accompanied by adequate tissue perfusion (ScvO_2_ 82.2 (78.2–85.2)%), high cortisol level (674 (670–780 nmol/l) (r = 0.42), while the insulin level varied within a wide range (510 (210–830) pmol/l).

### Group 3 (non-survivors)

In this group we included the patients who dead from sepsis after a relatively long time (more than 10 days) from their ICU admission. We remind that the patients who dead from the shock in the “ebb phase” have been excluded from this research. The non-survival patients had the highest TBSA third degree burns, and the most of the patients had inhalation injury. Therefore, all patients from this group were intubated in the first day. The APACHE II score, ScvO_2_, and glucose at admission were slightly higher than those for Group 2, while both the cortisol and insulin levels were lower. In the “flow phase”, three days after trauma, both mean daily glycemia and DELTA were higher than for any of the survival groups, while the minimum daily glucose level was smaller and the maximum level was approximately the same as for Group 2. During the ICU stay, both the cortisol (323.5 (92.2–779.2) nmol/l) and insulin (175.3 (112.1–667.8) pmol/l) concentrations in the blood varied within a wide range. No notable relation was found between TBSA burns (even if the average increases, the overlap in the ranges prevents us to give any prediction), APACHE II scores, mortality rate, ICU stay, and glucose at admission. However, we found strong association between mortality and increasing DELTA (r = +0.61); typically, DELTA began to grow up several days before death.

To compare the possibility for sepsis prognosis using DELTA and mean daily glucose, we performed a multiple regression analysis by calculating coefficients of statistical modeling, since the distributions of both the mean daily glucose and DELTA were normal. Our analysis has shown a high correlation of DELTA with burn severity (r = 0.7), while the correlation of the mean daily glucose was much lower (r = 0.36). Almost 100% probability for sepsis development occurs when the mean daily glucose exceeds 8 mmol/l and DELTA >6 mmol/l. Instead, when the disease outcome was favorable, the mean daily glucose did not exceed 6 mmol/l and DELTA was between 2 and 4 mmol/l.

Thus, both the time series and statistical analyses confirmed that DELTA is a sensitive and specific predictor of burn severity and illness outcome, better than hyperglycemia and mean daily glucose.

### DELTA amplification

**Figure 4:**
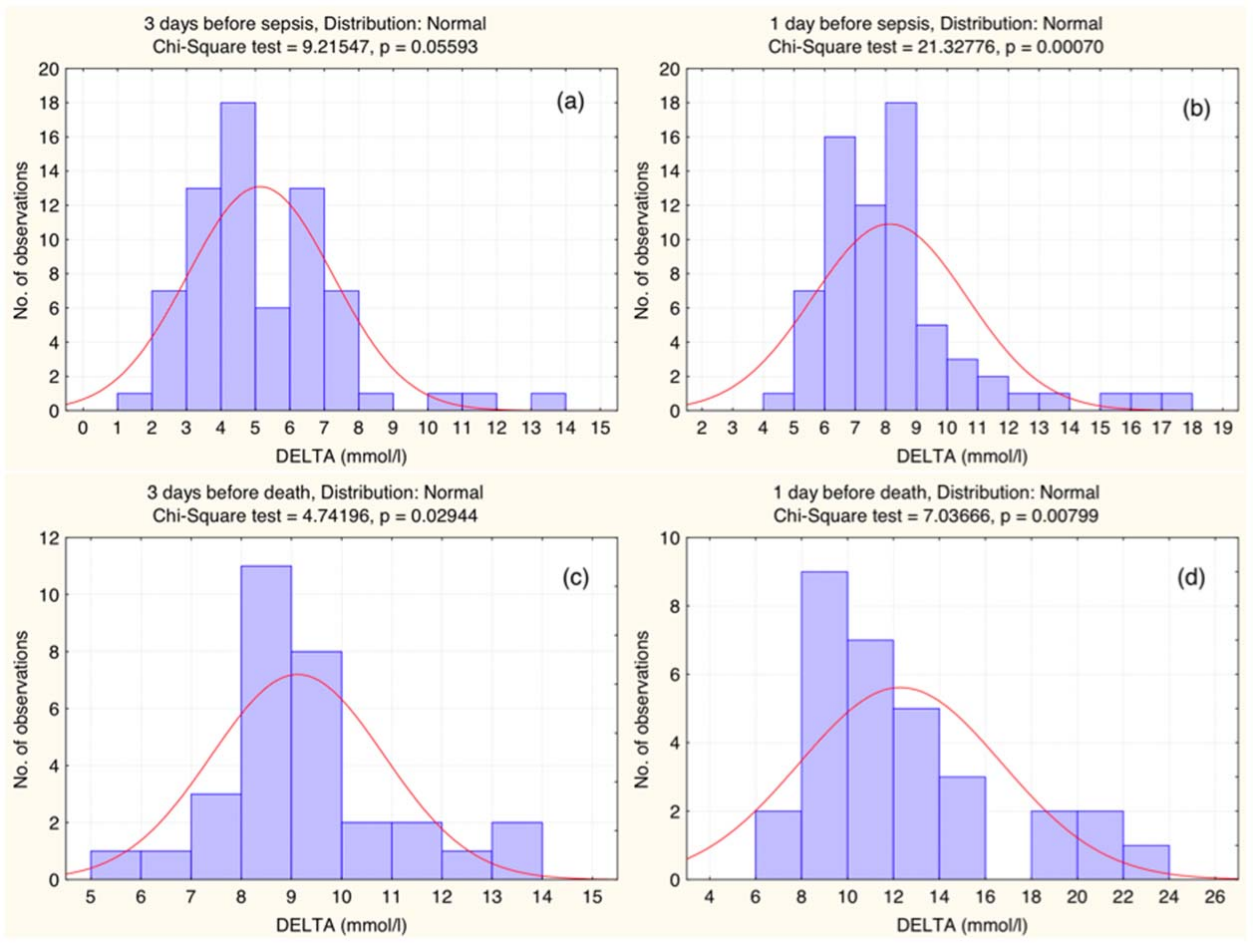
Distribution of DELTA before sepsis and death. (a) Three and (b) one day before sepsis. (c) Three and (d) one day before death. The solid lines are Gaussian approximations.

To study DELTA amplification before the critical point, we performed a separate analysis of the BG measurements of patients from Group 2 and Group 3. From the former group, we included in the analysis only the DELTA data obtained during five days preceded the date when sepsis was identified in the blood, and for the letter group, during five days before death. Statistical analysis yields normal distribution of DELTA in each of these days. The DELTA distributions one day and three days preceded sepsis and death are shown in Figs. 4(a,b) and 4(c,d), respectively. Since the distributions are normal, they are approximated by the Gaussian curves. One can see that during three days the most probable DELTA increased from 5 to 8 mmol/l before sepsis, and from 9 to 12 mmol/l before death. The DELTA data obtained one day before sepsis and one day before death, as well as the minimum and maximum daily glucose are included in Table 1. One can note a high difference from DELTA obtained during the whole ICU stay.

**Figure 5:**
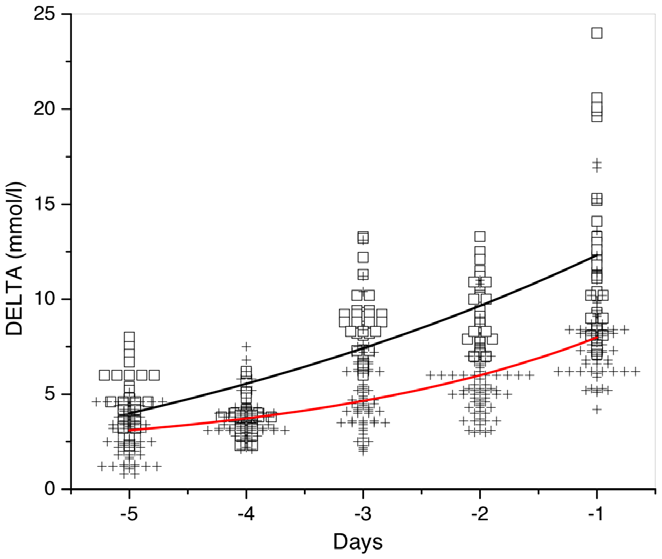
Increasing DELTA near critical point. Red crosses and black squares indicate, respectively, DELTA of survived and non-survived patients before sepsis and before death. Red and black curves are the exponential approximations with 2.6 and 5.6 growth rates, respectively.

A strong increase in DELTA before sepsis and death is clearly seen in Fig. 5, where we plot DELTA as a function of time anteceded these events. The fitting by the exponential function 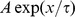 yields different growth rates for survival (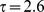) and non-survival (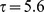) patients. While five days before sepsis or death, the difference in the mean DELTA between the two groups was very small (3 vs 4 mmol/l), approaching sepsis or death this difference became more and more pronounced (7.5 vs 12.5 mmol/l).

## Discussion

In the acute stress response, neuroendocrine stimulations with high levels of glucagon, catecholamines, glucocorticoids, and proinflammatory cytokines are important factors leading to hyperglycemia. Since at admission almost all severely burned patients presented hyperglycemia, we consider it is only a reflection of hemodynamic and respiratory failures during the burn shock and therefore admission glucose cannot be a prognostic value for septic complications. The counter-regulatory hormones tend to raise the BG level as a result of stimulation of hepatic gluconeogenesis and glycogenolysis and peripheral insulin resistance for redirecting energy supply to vital organs [49]. Hyperglycemia immediately after trauma (“ebb phase”) in all patients is associated with hypovolemia, hypoxia, a decrease in oxygen delivery, and tissue perfusion. (Note that the patients who died due to a burn shock in the first three days after trauma were excluded from this research). We believe that at this stage hyperglycemia is due to both the high glucose production accompanied by hypercortisolemia and low glucose utilization because of a lack of oxygen delivery and insulin resistance; this phenomenon is evidently associated with an adaptive mechanism for survival [46], [48]. The data reveal significant changes in glucose measurements (daily mean, minimum, maximum, and DELTA) between non-septic survival, septic survival, and non-survival patients in “flow phase”. We found that non-survivors had higher values for maximum, mean, and DELTA, while presenting episodes of hypoglycemia. These results are in agreement with the findings of Ligtenberg and coauthors [50], who suggested that the mean glucose level is not an independent risk factor for mortality.

Our analysis has shown that an increase in DELTA above 6 mmol/l is very dangerous because this may indicate on sepsis and forthcoming death (see Figs. 2 and 3). The absolute majority of the survived patients on the day when sepsis was detected had DELTA >6 mmol/l, while the majority of non-survivors had DELTA >8 mmol/l one day before death (see Table 1). Our observation that increasing glucose variability is independently associated with sepsis is in agreement with the results of other researchers [32], [57]. For instance, in the Waeschle€s et al. [57] study, patients with severe sepsis and septic shock, who were treated with intensive insulin therapy had a high risk of hypoglycemia and hyperglycemia; every patient who died had a SD >1.11 mmol/l. Monnier and Colette [20] pointed out that glucose variability should be one of the treatment targets of the glycemic disorders encountered in patients with type-2 diabetes. We agree with their opinion and believe that this statement should be extended to critically ill patients.

We did not find association of glucose variability with doses of intravenous insulin and glucose. Contrary to previously stated by some other authors [51], we did not find association of the peak glucose levels with the use of glucocorticoids, nutritions, and intravenous glucose. Every one of the patients who died had septic complications (hepatic, adrenal, renal, dysfunction). Dysfunctions of neoglucogenic organs (liver and kidney) can lead to impaired responsiveness of counter-regulatory hormones and episodes of hypoglycemia [52], making normoglycemia difficult to achieve. It was rather difficult to stabilize the glycemia level within a permissible range (between 3.5 and 8.0 mmol/l), and one of the main reasons is hypoglycemia [53]. The episodes of hypoglycemia were evident in 8.7% and 14.5% cases in survival and non-survival septic patients, respectively, and never observed in patients without sepsis, this is in agreement with the results reported in the literature [54]–[57]. Nevertheless, large DELTA was detected in majority cases in septic patients; 91.3% of survivors and 85.5% of non-survivors had DELTA >4.0 mmol/l even though mean glucose felt into an acceptable range.

Our preliminary results obtained with permanent glucose monitoring, as well as the results of other researchers [39], [58] testify that the BG oscillations have a random character, different periods ranging from 10 min to several hrs have been found. While approaching severe sepsis with a septic shock, DELTA increased in spite of unavailing therapeutic efforts to maintain this homeostatic value within its physiological region. Our analysis suggests a simple explanation for such a behavior as a clinical manifestation of the widespread phenomenon of fluctuation amplification near the onset of a dynamical instability observed previously in many other dynamical systems [4], [8], [59]. The results of this study confirm that strong DELTA amplification anticipates sepsis and forthcoming death. The rate of the DELTA growth during several days may serve as an indicator of the illness severity.

In conclusion, to give a more accurate prediction of the illness development, one has also to take into account other vital homeostatic values, such as cortisol and insulin levels, body temperature, pH, etc. Their fluctuations should also be considered as an important diagnostic tool to predict how a disease will end. More efficient prognosis of the disease outcome requires more frequent (at least every hour) or continuous monitoring of BG and other homeostatic values in order to perform complex analysis of their dynamics, including Fourier spectra, phase spaces, Lyapunov exponents, etc.
